# miR-301b-3p Regulates Breast Cancer Cell Proliferation, Migration, and Invasion by Targeting NR3C2

**DOI:** 10.1155/2021/8810517

**Published:** 2021-01-21

**Authors:** Yaohua Fan, Yan Li, Yuzhang Zhu, Guiping Dai, Dongjuan Wu, Zhenzhen Gao, Lei Zhang, Danying Xu

**Affiliations:** ^1^Department of Oncology, The Second Affiliated Hospital of Jiaxing University, Jiaxing, Zhejiang Province, China; ^2^Department of Breast Surgery, The Second Affiliated Hospital of Jiaxing University, Jiaxing, Zhejiang Province, China

## Abstract

**Objectives:**

Breast cancer is the most common malignant tumor among females, and miRNAs have been reported to play an important regulatory role in breast cancer progression. This study aimed to explore the function and underlying molecular mechanism of miR-301b-3p in breast cancer.

**Methods:**

Differential analysis and survival analysis were performed based on the data accessed from the TCGA-BRCA dataset for identification of the target miRNA. Bioinformatics analysis was conducted to predict the downstream target gene of the miRNA. Real-time quantitative PCR was carried out to detect the expression of miR-301b-3p and nuclear receptor subfamily 3 group C member 2 (NR3C2). Western blot was used to assess the protein expression of NR3C2. Cell counting kit-8 assay was performed to evaluate the proliferation of breast cancer cells. Transwell assay was conducted to determine the migratory and invasive abilities of breast cancer cells. Dual-luciferase reporter assay was employed to verify the targeting relationship between miR-301b-3p and NR3C2.

**Results:**

miR-301b-3p was elevated in breast cancer cell lines and promoted cell proliferation, migration, and invasion in terms of its biological function in breast cancer. NR3C2 was validated as a direct target of miR-301b-3p via bioinformatics analysis and dual-luciferase reporter assay, and NR3C2 was downregulated in breast cancer cell lines. The rescue experiment indicated that NR3C2 was involved in the mechanism by which miR-301b-3p regulated the malignant phenotype of breast cancer cells.

**Conclusion:**

The present study revealed for the first time that miR-301b-3p could foster breast cancer cell proliferation, migration, and invasion by targeting NR3C2, unveiling that miR-301b-3p is a novel carcinogen in breast cancer.

## 1. Introduction

As a complicated and common disease, breast cancer is the fifth leading cause, resulting in cancer-associated death worldwide. It was estimated that there were 2,088,849 newly confirmed cases and 626,679 deaths of breast cancer in 2018, and the number is continually increasing [1]. Meanwhile, recurrence and tumor metastasis often occur to patients, which intensify the threat that breast cancer poses and triggers considerable economic burden globally [2]. As a highly heterogeneous disease, the etiology of breast cancer is complex, including various environmental factors and gene alteration that sparks changes in cell process [3, 4]. With regard to the clinical treatment of breast cancer, there are certain side effects of the current therapeutic strategies. For example, chemotherapy is mainly designed to prolong the patients' survival time, but it is unable to distinguish normal cells from cancer cells leading to limited curative effect [5]. Accordingly, it is urgent for researchers to make further progress concerning prevention, diagnosis, and treatment by starting from the biological mechanisms so as to improve the prognosis and survival of breast cancer sufferers [6].

In recent years, the regulatory role of miRNAs in the occurrence, development, and metastasis of breast cancer has aroused immense interest among people [7]. The level of sequence complementarity between miRNA and its target mRNA determines the inhibitory mechanism of protein expression. Due to the short miRNA-mRNA binding site, a single miRNA is able to target multiple mRNAs and regulate their functions via various approaches [8, 9]. For instance, miR-1207-5p is capable of regulating the expression of CDKN1A and CDKN1B to foster breast cancer cell proliferation and cell cycle progression by interacting with STAT6 [10]. miR-497 suppresses breast cancer metastasis and immune response or tumor immune escape by targeting CD274 [11]. Consequently, as the key factor of signaling cascades, miRNAs are a type of underlying biomarkers for clinical diagnosis and can be applied to the treatment of breast cancer as a therapeutic target and tool.

This study verified that miR-301b-3p might be the unfavorable factor of the prognosis of breast cancer via bioinformatics analysis. Research has suggested that miR-301b-3p as an oncogene plays an important role in various human cancers, such as high-grade ovarian serous tumor [12], gastric cancer [13], and non-small-cell lung cancer [14], and seriously affects patients' prognosis. However, there have been no studies about the role of miR-301b-3p in breast cancer. As a result, the present study aimed to validate whether miR-301b-3p regulated breast cancer progression and explore its functional mechanism so as to provide new ideas and strategies for the treatment of breast cancer.

## 2. Materials and Methods

### 2.1. Bioinformatics Analysis

Expression profiles including miRNA (normal: *n* = 104 and tumor: *n* = 1,103) and mRNA (normal: *n* = 113 and tumor: *n* = 1,109) of breast cancer were downloaded from the TCGA-BRCA dataset (https://portal.gdc.cancer.gov/), and differential analysis was conducted to screen the differentially expressed miRNAs (DE_miRNAs) and differentially expressed mRNAs (DE_mRNAs) using the “edgeR” package (|logFC|>2, *p*adj<0.01). Survival analysis was performed on DE_miRNAs based on the clinical data of samples to confirm the target miRNA. miRDB (http://mirdb.org/), TargetScan (http://www.targetscan.org/vert_71/), miRTarBase (http://mirtarbase.mbc.nctu.edu.tw/php/index.php), and starBase (http://starbase.sysu.edu.cn/) databases were employed to predict the target genes of miR-301b-3p, which were then intersected with downregulated DE_mRNAs in the TCGA-ESCA dataset, and the ultimate target mRNA was obtained.

### 2.2. Cell Culture

Normal human breast cell line Hs 578Bst (no. 3131C0001000200016), human breast cancer cell lines MCF7 (no. 3111C0001CCC000013), MDA-MB-231 (no. 3111C0001CCC000014) and SK-BR-3 (no. 3111C0001CCC000085), and 293T cell line (no. 3111C0001CCC000091) which was used for dual-luciferase reporter assay were all accessed from National Infrastructure of Cell Line Resource (Beijing, China). Hs 578Bst and 293T cells were cultured in Dulbecco's Modified Eagle's Medium (DMEM; Hyclone, USA) supplemented with 10% fetal bovine serum (FBS). Breast cancer cells were maintained in RPMI 1640 (Hyclone, USA) medium containing 10% FBS. All the cells were incubated in an incubator of 5% CO_2_ at 37°C.

### 2.3. Cell Transfection

miR-301b-3p mimic and its corresponding negative control (NC) mimic were designed and synthesized by GenePharma (Shanghai, China). Nuclear receptor subfamily 3 group C member 2 (NR3C2) overexpression vector was constructed by HonorGene (Shanghai, China) with pcDNA3.1 empty vector as control. MDA-MB-231 cells were seeded into a 6-well plate and kept for 12 h. Thereafter, cells were cotransfected with miR-301b-3p mimic, NR3C2 overexpression vector, and their corresponding NC using Lipofectamine 2000 reagent (Invitrogen, CA, USA) according to the manufacturer's instructions.

### 2.4. Real-Time Quantitative PCR (qRT-PCR)

Total RNA was extracted from cells using TRIzol reagent (Invitrogen) according to the manufacturer's instructions. Then, RNA was transcribed into complementary DNA (cDNA) by using the PrimeScript Reverse Transcription Assay Kit (TaKaRa, Liaoning, China). qRT-PCR was performed on ABI StepOnePlus™ real-time PCR System (Applied Biosystems, USA) using SYBR Green Mix (Toyobo, Japan). U6 and GAPDH were used as the internal references of miR-301b-3p and NR3C2. The quantitative value was expressed using the 2^−ΔΔCt^ method. The primer sequences are listed in Table 1.

### 2.5. Western Blot

Total proteins were extracted from cells using RIPA lysis buffer (Beyotime, China), and the concentration of proteins was measured by BCA protein assay kit (Thermo Fisher, USA). Protein samples were separated on sodium dodecyl sulphate-polyacrylamide gel electrophoresis (SDS-PAGE) and transferred onto the polyvinylidene fluoride (PVDF; EMD Millipore, Billerica, MA, USA) membranes with 5% nonfat milk. Afterwards, the membranes were incubated with primary anti-NR3C2 (1 μg/ml, ab64457, Abcam, UK) and anti-GAPDH (1 : 1000, ab8245, Abcam, UK) at 4°C, followed by hybridization with horseradish peroxidase- (HRP-) conjugated secondary antibody LgG H&L (HRP) (1 : 2000, ab6721, Abcam, UK). WesternLightning Plus-ECL (PerkinElmer) was employed for protein bands visualization, and the LAS-3000 Luminescent Image Analyzer (Fujifilm) was applied for bands analysis.

### 2.6. Cell Counting Kit-8 (CCK-8) Assay

MDA-MB-231 cells (2 × 10^3^ cells/well) were inoculated into a 96-well plate. CCK-8 assay kit (Dojindo Laboratory, Tokyo, Japan) was employed to assess cell proliferation. At 0 h, 24 h, 48 h, and 72 h after transfection, each well was added with 10 *μ*l CCK-8 solution and cells were incubated for consecutive 2 h. At last, the absorbance of each well at 450 nm was identified by using a microplate reader (Bio-Rad Instruments, Hercules, CA, USA).

### 2.7. Transwell Assay

Transwell inserts (8 *μ*m pore size; Corning, USA) were used for assessing cell migration and invasion. For migration assay, 5 × 10^4^ MDA-MB-231 cells were inoculated into the upper chamber, while the lower chamber was filled with 600 *μ*l precooled DMEM supplemented with 10% FBS to stimulate cell migration. After cells were cultured for 48 h, invasive cells in the lower chambers were fixed, stained, washed, dried, and finally observed and photographed under a microscope (100 × , Leica Inc., Wetzlar, Germany). The experiment was repeated three times. The procedures of the invasion assay were basically the same as that of the migration assay, except that the upper chamber needed to be precoated with 50 *μ*l Matrigel (1 : 8, Yepsen, Shanghai).

### 2.8. Dual-Luciferase Reporter Assay

cDNA fragments of the NR3C2 3′UTR containing the miR-301b-3p binding site were amplified by PCR and inserted into the pGL3 luciferase vector (Promega, Madison, WI, USA), contributing to the construction of the wild type (WT) NR3C2 (NR3C2-WT). The mutant (MUT) NR3C2 (NR3C2-MUT) was constructed using the fast site-directed mutagenesis kit (Agilent, Roseville City, CA, USA). 293T cells were cotransfected with NR3C2-WT or NR3C2-MUT reporter vector and miR-301b-3p mimic or NC mimic using Lipofectamine 2000. 48 h later, the luciferase activity was assessed using the dual-luciferase reporter assay kit (Promega).

### 2.9. Statistical Analysis

Each experiment was repeated independently at least three times. All statistical analyses were performed using GraphPad Prism 8.0 (GraphPad Software, Inc., La Jolla, CA) software. All the data were expressed as mean ± standard deviation (SD), and differences between two groups were analyzed by Student's *t*-test. *p* < 0.05 was considered statistically significant.

## 3. Results

### 3.1. miR-301b-3p Is Upregulated in Human Breast Cancer

**Figure 1:**
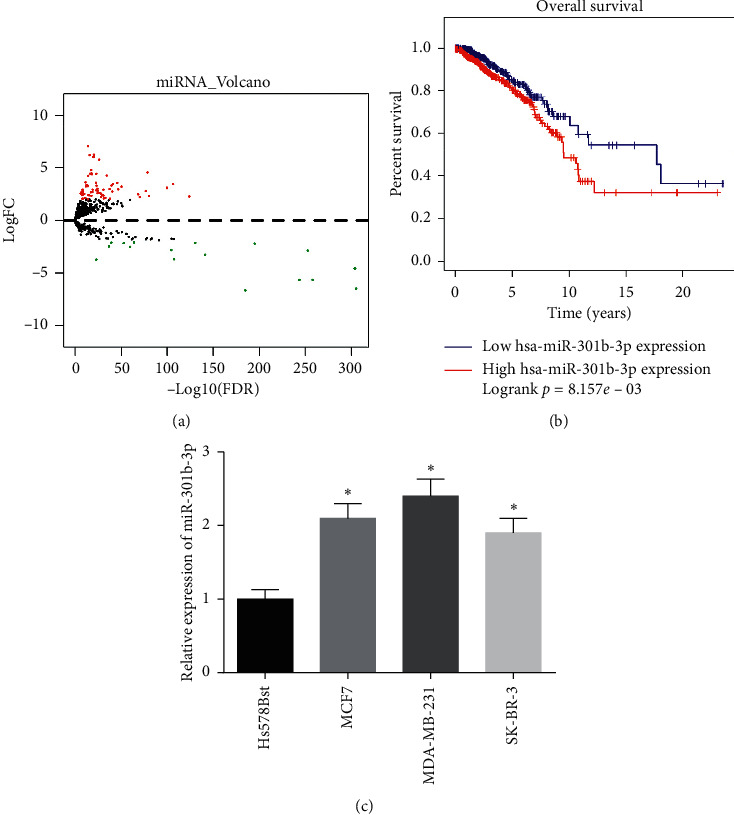
Upregulation of miR-301b-3p in human breast cancer: (a) differential gene volcano map of miRNA in TCGA-BRCA (red indicates upregulated miRNA, and green indicates downregulated miRNA); (b) survival curve of miR-301b-3p gene (red curve indicates high expression, and blue curve indicates low expression); (c) the expression level of miR-301b-3p in normal breast cells and breast cancer cell lines (^*∗*^,*p* < 0.05).

In order to preliminarily verify the miRNA which might regulate breast cancer progression, differential analysis was performed based on the miRNA expression profiles accessed from the TCGA-BRCA dataset and 86 DE_miRNAs were obtained (Figure 1(a)). Survival analysis indicated that the survival time of patients with high miR-301b-3p expression was markedly shorter than that of patients with low miR-301b-3p expression; hence, miR-301b-3p was likely to be the disadvantageous prognostic factor of breast cancer (Figure 1(b)). Besides, miR-301b-3p is considered to be an oncogene in many researches [15, 16]. Accordingly, we detected its expression in breast cancer cells (Figure 1(c)) and found that its expression in breast cancer cells MCF7, MDA-MB-231, and SK-BR-3 was noticeably higher than that in normal breast cell Hs 578Bst. miR-301b-3p was most significantly differentially expressed in MDA-MB-231 cells; hence, MDA-MB-231 cell line was chosen for follow-up experiment.

### 3.2. Upregulation of miR-301b-3p Promotes the Proliferation, Migration, and Invasion Abilities of Breast Cancer Cells

**Figure 2:**
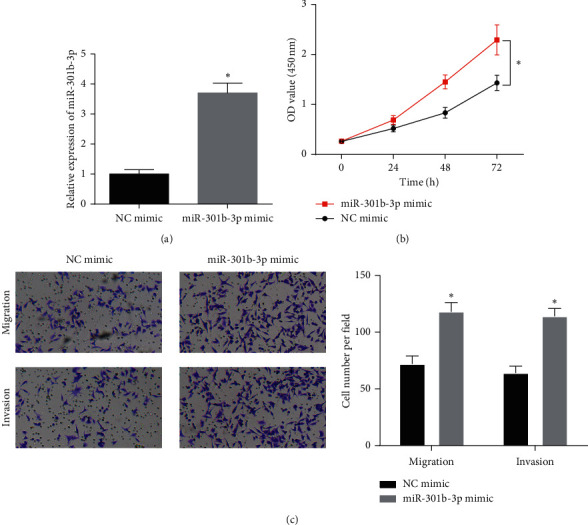
Upregulation of miR-301b-3p fosters the proliferation, migration, and invasion abilities of breast cancer cells. (a) qRT-PCR was used to detect the transfection efficiency of miR-301b-3p mimic. The effect of miR-301b-3p overexpression on breast cancer cell viability was detected by (b) CCK-8 assay. (c) Transwell assay was performed to assess the effect of miR-301b-3p overexpression on breast cancer cell migration and invasion abilities (100×) (^*∗*^*p* < 0.05).

To further investigate whether miR-301b-3p regulated breast cancer progression, CCK-8 assays were performed to assess breast cancer cell proliferation and transwell assay was conducted to evaluate the migration and invasion. miR-301b-3p mimic and NC mimic were transiently transfected into MDA-MB-231 cells, and qRT-PCR was used to detect the transfection efficiency (Figure 2(a)). CCK-8 assay uncovered that compared with the control group, the viability of MDA-MB-231 cells transfected with miR-301b-3p mimic was significantly higher (Figure 2(b)). Additionally, transwell assay showed that compared with the control group, the numbers of migratory and invasive MDA-MB-231 cells were remarkably increased in the miR-301b-3p mimic group (Figure 2(c)). Collectively, the above results demonstrated that miR-301b-3p could regulate breast cancer progression as an oncogene.

### 3.3. NR3C2 Is a Direct Target of miR-301b-3p

**Figure 3:**
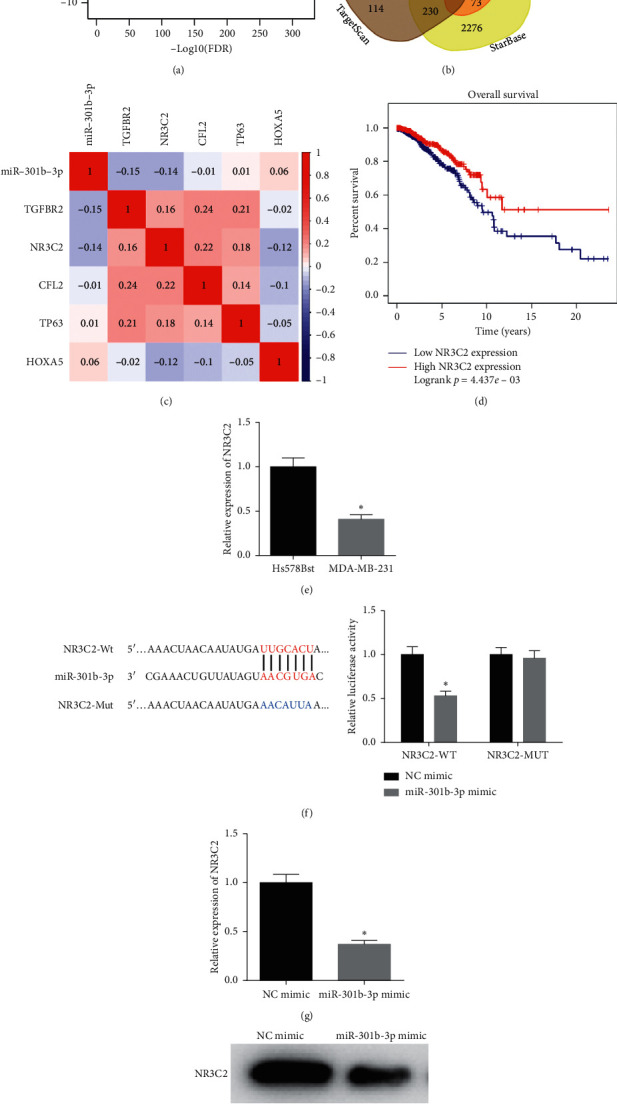
NR3C2 is a direct target of miR-301b-3p: (a) volcano plot of DE_mRNAs in the TCGA-BRCA dataset (red and green represent upregulated and downregulated DE_mRNAs, respectively); (b) Venn diagram of downregulated DE_mRNAs in the TCGA-BRCA dataset and the predicted target mRNAs; (c) correlation analysis of miR-301b-3p and the 5 overlapping mRNAs; (d) survival analysis of NR3C2 (red and blue represent high and low expressions, respectively); (e) qRT-PCR was performed to detect NR3C2 expression in breast cancer cell MDA-MB-231 and normal breast cell Hs 578Bst; (f) binding site sequence of miR-301b-3p on NR3C2 3′UTR was predicted by TargetScan database, and dual-luciferase reporter assay was conducted to verify the targeting relationship between them; the effect of miR-301b-3p overexpression on NR3C2 mRNA and protein expression was evaluated by (g) qRT-PCR and (h) western blot. ^*∗*^*p* < 0.05.

Previously, we had unveiled that miR-301b-3p as an oncogene could regulate breast cancer cell proliferation, migration, and invasion. Next, the molecular mechanism by which miR-301b-3p fostered breast cancer progression was explored and validated. 2,146 DE_mRNAs were obtained using differential analysis (Figure 3(a)). miRDB, TargetScan, miRTarBase, and starBase databases were employed to predict the target genes of miR-301b-3p, which were then intersected with downregulated DE_mRNAs in the TCGA-ESCA dataset. 5 DE_mRNAs (TGFBR2, NR3C2, TP63, HOXA5, and CFL2) which had binding sites with miR-301b-3p were obtained (Figure 3(b)), where the correlation analysis found that NR3C2 is highly correlated with miR-301b-3p, and survival analysis shows that patients with high NR3C2 expression have a better prognosis (Figures 3(c)–3(d)). qRT-PCR suggested that the expression of NR3C2 in breast cancer cell MDA-MB-231 was markedly lower than that in normal breast cell Hs 578Bst (Figure 3(e)). The TargetScan database was used to predict the binding site sequence of miR-301b-3p on NR3C2 3′UTR. Dual-luciferase reporter assay demonstrated that overexpressing miR-301b-3p could significantly inhibit the luciferase activity in the NR3C2-WT group but showed no noticeable effect on that in the NR3C2-MUT group (Figure 3(f)). Additionally, qRT-PCR and western blot indicated that NR3C2 mRNA and protein expression were remarkably reduced upon miR-301b-3p overexpression (Figures 3(f) and 3(g)). Collectively, the above results unveiled that NR3C2 was a direct target of miR-301b-3p in breast cancer.

### 3.4. miR-301b-3p Promotes Breast Cancer Cell Proliferation, Migration, and Invasion by Targeted Silencing NR3C2

**Figure 4:**
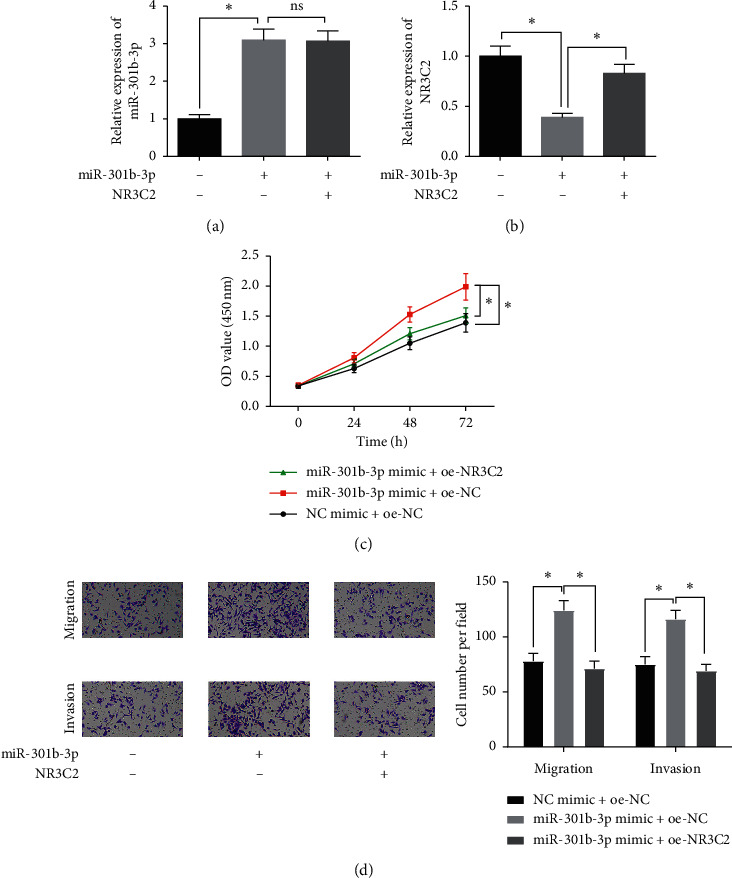
miR-301b-3p regulates the malignant progression of breast cancer cells by targeting NR3C2. (a-b) qRT-PCR was performed to assess the expression of miR-301b-3p and NR3C2 in the three different transfection groups; cell viability and proliferation were evaluated by (c) CCK-8 assay in the three groups; (d) transwell assay was conducted to determine the invasive and migratory abilities in the three groups (100×). ^*∗*^*p* < 0.05.

The carcinogenic role of miR-301b-3p in breast cancer progression and the targeting relationship between miR-301b-3p and NR3C2 had been verified through a series of experiments. The rescue experiment was carried out to confirm whether miR-301b-3p facilitated breast cancer cell proliferation, migration, and invasion abilities by directly targeting NR3C2. MDA-MB-231 cells were divided into 3 groups for transfection: NC mimic + oe-NC, miR-301b-3p mimic + oe-NC, and miR-301b-3p mimic + oe-NR3C2. To better perform the subsequent *in vitro* functional experiments, firstly we detected the expression of miR-301b-3p and NR3C2 in cells of the above 3 groups, finding that overexpressing miR-301b-3p markedly inhibited NR3C2 mRNA expression, while the restoration of NR3C2 showed no effect on the expression of miR-301b-3p (Figures 4(a) and 4(b)). CCK-8 assay revealed that compared with the NC mimic + oe-NC group, upregulation of miR-301b-3p significantly facilitated the viability and growth ability of MDA-MB-231 cells. Compared with the miR-301b-3p mimic + oe-NC group, MDA-MB-231 cell proliferation capacity was restored to some extent with the restoration of NR3C2 expression (Figure 4(c)). In the transwell assay, migratory and invasive abilities of MDA-MB-231 cells were remarkably enhanced by overexpressing miR-301b-3p, which was markedly suppressed with the restoration of NR3C2 expression (Figure 4(d)). Taken together, the promoting effect of miR-301b-3p on the proliferation, migration, and invasion of breast cancer cells could be repressed by overexpressing NR3C2 to a certain degree. In other words, miR-301b-3p facilitated the malignant progression of breast cancer by targeted downregulating NR3C2.

## 4. Conclusion

The reason behind the easy recurrence of breast cancer sufferers after treatment is that the conventional therapeutic strategies lack sensitivity and specificity, which indicates a high demand for systematic treatment. On the contrary, accumulating evidence has suggested that miRNAs are important regulatory factors of cancer progression and they can be used as a novel diagnostic tool as well as a valuable therapeutic approach [17, 18]. In the present study, we discovered that miR-301b-3p was elevated in breast cancer cell lines and markedly affected patients' prognosis. Therefore, we speculated that miR-301b-3p might be closely related to the malignant progression of breast cancer. In order to verify this speculation, we constructed the miR-301b-3p overexpression model and found that upregulation of miR-301b-3p significantly raised the growth tendency and metastatic potential of breast cancer cells.

miR-301b-3p is a newly discovered miRNA. Despite the few studies about it, its promoting effect on the malignant progression of various human cancers has been reported gradually, which demonstrates that miR-301b-3p usually functions as an oncogene. In the prostate cancer (PC), hypoxia-induced miR-301b-3p is likely to foster PC cell proliferation, migration, and invasion through targeting LRP1B [15]. Man et al. [19] reported that USP13 is a direct target of miR-301b-3p, and it can be downregulated by miR-301b-3p overexpression, leading to decreased protein expression of PTEN, consequently promoting the occurrence of bladder cancer. Before these studies, there had been no research unveiling the expression and the role of miR-301b-3p in breast cancer. Nonetheless, the current studies support our discovery to some degree that miR-301b-3p as an oncogene regulates breast cancer cell proliferation, migration, and invasion. In this study, we found that upregulated miR-301b-3p in breast cancer might be the unfavorable factor of prognosis via bioinformatics analysis, indicating that miR-301b-3p was likely to be closely associated with the malignant progression of breast cancer. In vitro experiments also clearly revealed the promoting effect of miR-301b-3p on the proliferation, migration, and invasion of breast cancer cells. These findings immensely fill the vacancy of miR-301b-3p researches in breast cancer.

Additionally, we discovered that NR3C2 was a direct target of miR-301b-3p in breast cancer. Yang et al. [20] once mentioned the targeting relationship between miR-301b-3p and NR3C2 in a report about pancreatic cancer. As a mineralocorticoid receptor (MR) gene coding MR, NR3C2 is a transcription factor which plays a crucial role in regulating electrolyte balance [21]. It has been reported that NR3C2 is poorly expressed in oral squamous cell carcinoma [22], clear cell renal cell carcinoma [23], and acute myeloid leukemia [24] and NR3C2 overexpression inhibits the proliferation, migration of tumor cells, and angiogenesis. In this study, we found that NR3C2 was underexpressed in breast cancer cell lines and overexpressing NR3C2 suppressed the proliferation, migration, and invasion of breast cancer cells, which was consistent with the researches of NR3C2 in other cancers. In addition, researchers have reported that low level of MR predicts the poor prognosis of patients with lung cancer and colon cancer [25, 26]. We also discovered that downregulation of NR3C2 indicated the unfavorable prognosis of breast cancer sufferers, which backed up the possibility of NR3C2 serving as a marker for the prognosis of breast cancer patients.

In conclusion, we revealed the expression pattern and roles of miR-301b-3p and NR3C2 in breast cancer cells and confirmed that miR-301b-3p promoted breast cancer cell proliferation, migration, and invasion by targeted silencing NR3C2, which helps to provide research support for the design of targeted drugs for breast cancer. However, this study is partially limited as we did not make a deeper exploration into the downstream signaling pathway by which NR3C2 repressed the malignant progression of breast cancer. Consequently, we will investigate the relatively complete regulatory axis of miR-301b-3p/NR3C2/signaling pathway in the following researches in order to improve the treatment status of breast cancer.

## Figures and Tables

**Table 1:** Primer sequences for qRT-PCR.

Gene	Prime sequence (5′-3′)
U6	Forward: CTCGCTTCGGCAGCACATA
	Reverse: AACGATTCACGAATTTGCGT
miR-301b-3p	Forward: CAGTGCTCTGACGAGGTTG
	Reverse: TGTCCCAGATGCTTTGACA
GAPDH	Forward: GAGAAGGCTGGGGCTCATTT
	Reverse: AGTGATGGCATGGACTGTGG
NR3C2	Forward: GAGCCCATTGCTAGCA
	Reverse: GCCCTGCTGGAATCACTCT

## Data Availability

The data used to support the findings of this study are included within the article. The data and materials in the current study are available from the corresponding author on reasonable request.

## Additional Points

1. miR-301b-3p was upregulated in breast cancer and could foster cell proliferation, migration, and invasion. 2. NR3C2 was downregulated in breast cancer and inhibited cell proliferation, migration, and invasion. 3. miR-301b-3p facilitated the malignant progression of breast cancer by targeted downregulating of NR3C2.
